# Investigating the Circulation of Ebola Viruses in Bats during the Ebola Virus Disease Outbreaks in the Equateur and North Kivu Provinces of the Democratic Republic of Congo from 2018

**DOI:** 10.3390/pathogens10050557

**Published:** 2021-05-04

**Authors:** Audrey Lacroix, Placide Mbala Kingebeni, Simon Pierre Ndimbo Kumugo, Guy Lempu, Christelle Butel, Laetitia Serrano, Nicole Vidal, Guillaume Thaurignac, Amandine Esteban, Daniel Mukadi Bamuleka, Jacques Likofata, Eric Delaporte, Jean-Jacques Muyembe Tamfum, Ahidjo Ayouba, Martine Peeters, Steve Ahuka Mundeke

**Affiliations:** 1 TransVIHMI (Recherches Translationnelles sur VIH et Maladies Infectieuses), Université de Montpellier, Institut de Recherche pour le Développement, INSERM, 34394 Montpellier, France; audrey.lacroix@ird.fr (A.L.); christel.butel@ird.fr (C.B.); laetitia.serrano@ird.fr (L.S.); nicole.vidal@ird.fr (N.V.); guillaume.thaurignac@ird.fr (G.T.); amandine.esteban@ird.fr (A.E.); eric.delaporte@ird.fr (E.D.); ahidjo.ayouba@ird.fr (A.A.); 2 Institut National de Recherche Biomédicale (INRB), 1197 Kinshasa, Democratic Republic of the Congo; placidembala@inrb.net (P.M.K.); simonp_ndimbok@yahoo.fr (S.P.N.K.); kajosmpiana@gmail.com (G.L.); jjmuyembet@gmail.com (J.-J.M.T.); 3 Service de Microbiologie, Cliniques Universitaires de Kinshasa, 1197 Kinshasa, Democratic Republic of the Congo; drmukadi@gmail.com; 4 Institut National de Recherche Biomédicale (INRB), Goma, Democratic Republic of the Congo; 5 Laboratoire Provincial de Mbandaka, Equateur, Democratic Republic of the Congo; jacqueslikofata@gmail.com

**Keywords:** Ebola virus, bats, the Democratic Republic of the Congo

## Abstract

With 12 of the 31 outbreaks, the Democratic Republic of Congo (DRC) is highly affected by Ebolavirus disease (EVD). To better understand the role of bats in the ecology of Ebola viruses, we conducted surveys in bats during two recent EVD outbreaks and in two areas with previous outbreaks. Dried blood spots were tested for antibodies to ebolaviruses and oral and rectal swabs were screened for the presence of filovirus using a broadly reactive semi-nested RT-PCR. Between 2018 and 2020, 892 (88.6%) frugivorous and 115 (11.4%) insectivorous bats were collected. Overall, 11/925 (1.2%) to 100/925 (10.8%) bats showed antibodies to at least one Ebolavirus antigen depending on the positivity criteria. Antibodies were detected in fruit bats from the four sites and from species previously documented to harbor Ebola antibodies or RNA. We tested for the first time a large number of bats during ongoing EVD outbreaks in DRC, but no viral RNA was detected in the 676 sampled bats. Our study illustrates the difficulty to document the role of bats as a source of Ebolaviruses as they might clear quickly the virus. Given the increasing frequency of EVD outbreaks, more studies on the animal reservoir are urgently needed.

## 1. Introduction

Since the first recognized Ebolavirus disease (EVD) outbreak in humans in 1976 in the Democratic Republic of Congo (DRC), a total of 31 outbreaks have now been recognized in Africa [1,2,3]. The majority remained limited in the number of infected individuals and in the geographic spread, but the outbreaks in West Africa (December 2013 to March 2016) and in Eastern DRC (August 2018 to June 2020) clearly showed that they can also become epidemic and infect thousands of individuals over large geographic areas [4]. In addition, the frequency of EVD outbreaks also increased over the last decades. With twelve outbreaks, DRC is one of the most affected countries. Since May 2018, the country has been continuously facing the circulation of the virus during four consecutive outbreaks [3,5,6,7]. The largest EVD outbreak occurred in the eastern provinces of North Kivu and Ituri over almost two years (July 2018–June 2020). It infected 3470 people and 2299 (66.2%) patients died [6,7]. The eleventh outbreak occurred in the Equateur province between June and November 2020, close to areas of the 2018 outbreak in the same province [2,7]. On the 7th February 2021, a new resurgence has been declared in North Kivu, involving 11 cases to date [3,8].

Except for a few recent outbreaks linked to a resurgence in humans [9,10], EVD outbreaks are most likely the result of independent zoonotic transmission event(s) and bats are considered as reservoir species [11,12,13]. Although there is no direct evidence of exposure to infected bats, two EVD outbreaks have been suspected to be linked to bats; i.e., in Luebo (DRC) in 2007 and the major outbreak in West Africa in 2013 [12,14]. Other findings increase the likelihood of the role of bats in the filovirus ecology. For example, the detection of another filovirus, Marburg virus (MARV), in bats across Africa (Uganda, RDC, Kenya, South Africa, Gabon, Zambia, Sierra Leone) [15,16,17,18,19,20,21,22,23,24,25]. The detection of other filoviruses in bats, such as Lloviu virus in Europe [26,27], filoviruses in bats from China [28,29] and the Philippines [30,31] or Bombali virus (BOMV) in insectivorous bats in Africa provides additional evidence that filoviruses, including Ebola virus, have a Chiropteran origin [32,33,34]. So far, MARV is the only pathogenic filovirus isolated from bats [18], and Egyptian fruit bats (*Rousettus aegyptiacus*), identified as the reservoir, seem to support virus replication with no apparent disease [35,36].

Zaire Ebola virus (EBOV) RNA and antibodies were detected in three frugivorous bat species (*Epomops franqueti*, *Hypsignathus monstrosus* and *Myonycteris torquata*) during EVD outbreaks in 2003 in Gabon and the Republic of Congo [13]. Surveys of bats in West, Central and East Africa have revealed the presence of antibodies to Ebolaviruses in at least eight frugivorous and one insectivorous species (*Mops condylurus*) [13,16,37,38,39,40]. Only a few studies have been performed on bats and ebolaviruses in DRC [40,41,42,43]. Our previous serological survey in the Western province of Bas Congo where no previous EVD outbreaks have been reported, investigated a total of 830 bats, including 428 frugivorous and 402 insectivorous bats, [40]. We identified antibodies in *E. helvum* but no viral RNA was detected.

Bats may represent a source of pathogen spillover into human populations in many African countries through their hunting and butchering for bushmeat or through indirect exposure to fruits contaminated by infected saliva, urine or faeces [44,45,46]. Nevertheless, intermediate amplifying hosts may also play a major role in the emergence of EVD outbreaks, as illustrated in Gabon and Ivory Coast where apes have been confirmed as the source of infection in several outbreaks [11,47]. Similar to humans, bats can also transmit their viruses to other mammals through contact with fruits contaminated with secretions or by hunting, for example, non-human primates (monkeys and bonobos) are reported to hunt bats [48,49].

Today, the ecology of Ebolaviruses is still poorly understood and the role of bats in outbreaks needs to be further clarified. In order to increase the chances to detect viral RNA and Ebola antibodies, we investigated the presence of RNA from filoviruses and Ebola antibodies in bats from DRC collected during the EVD outbreaks in the Equateur and North Kivu provinces and in other areas that have already experienced outbreaks.

## 2. Results

### 2.1. Samples and Species per Collection Site

**Figure 1:**
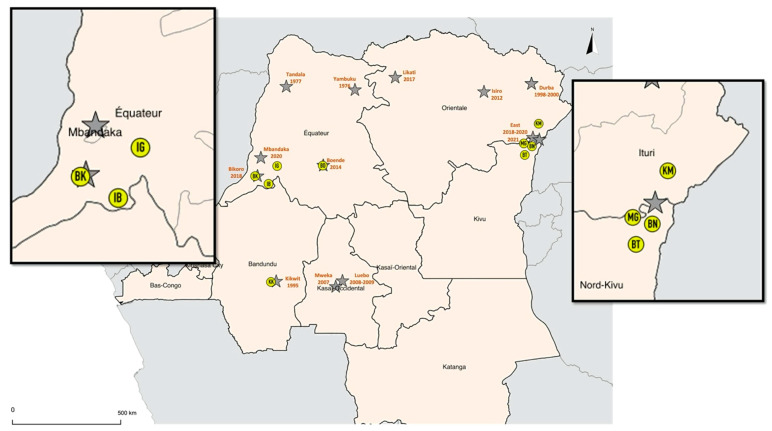
Collection sites of bat samples. Sites where bat samples were collected are highlighted with yellow circles. Grey stars represent filovirus outbreaks in DRC. Sites are abbreviated as follows: BK: Bikoro; IB: Iboko; IG: Ingende; BN: Beni; BT: Butembo; KM: Komanda; MG: Mangina; KK: Kikwit; BD: Boende.

Between February 2018 and October 2019, a total of 1007 bats were sampled in four provinces with previous and ongoing Ebola outbreaks (Figure 1, Table S1). Specifically, 288 bats were sampled during the ninth EVD outbreak in May–July 2018 in the Equateur province in three sites were Ebola cases were reported, i.e., Bikoro (BK), Ingende (IG) and Iboko (IB). A total of 453 bats were sampled between December 2018 and June 2019 during the tenth EVD outbreak in the Eastern provinces of North Kivu and Ituri, i.e., Komanda (KM), Mangina (MG), Beni (BN), Butembo (BT). Additionally, 98 and 168 bats were sampled around Kikwit (KK) in February 2018 and in Boende (BD) during September-October 2019, where the third and the seventh EVD outbreaks occurred in 1995 and 2014, respectively. Bats were captured in different ecological environments including 839 (83.3%) in anthropized sites, i.e., villages (14.4%) or cities (68.9%), and 168 in forest sites (16.6%).

The identification of the species was confirmed on 903 (90%) of the 1007 collected bats, 807 cytb and 96 12S sequences were obtained. The species identification of the remaining samples was extrapolated as described in the methods. For some insectivorous bats (*Molossidae*, *Hipposideridae*, *Vespertilionidae*, *Nycteridae*), identification was only possible at the genus level due to the lack of reference sequences in Genbank. Details on bat families, genera and species collected at each site are shown in Table 1. A total of 892 (88.6%) samples were from frugivorous bats belonging to 11 genera and 115 (11.4%) were from insectivorous bats, representing seven genera from five families. Overall, 48.4% of the samples were from female bats and 51.6% were from males, 97.4% were from adult bats and 2.6% were from juveniles.

### 2.2. Molecular Screening for Filoviruses

A total of 1121 swab samples from 676 bats, all from areas of ongoing outbreaks, were screened for the presence of viral RNA. Depending on the availability, oral and rectal swabs were both tested for 425 (63%) bats, and only oral and rectal swabs were tested for 56 (8%) and 193 (29%) bats, respectively (Table 2). Almost all the samples collected during the two EVD outbreaks were tested; i.e., 288/288 (100%) and 388/453 (86%) of the samples from the outbreak in the Equateur and North-Kivu/Ituri provinces, respectively. For 65 bats from the North Kivu outbreak, including all samples from Komanda (KM), swab samples were not available. None of the 1121 swabs tested positive for filovirus viral RNA. For 11 samples, PCR products at the expected size were observed, but MinIon sequencing did not reveal the presence of Ebolavirus sequences. The 11 samples were also tested using the VP35 ebolavirus specific RT-PCR, and were negative.

### 2.3. Serological Screening for Presence of Antibodies against Ebolaviruses

We tested dried blood spots (DBS) from 925/1007 (91.9%) bats for Ebolavirus antibodies. All samples from Boende and Kikwit and 99.6% and 82.1% of those collected during the outbreaks in Equateur Province and eastern provinces of DRC, respectively, were tested. As in our previous study, we used a range to express the number and percentages of reactive samples according to different cutoff calculations as described in the methods and shown in Supplementary Table S2 and Supplementary material 4. According to our previously defined positivity criteria, i.e., simultaneous reactivity for NP and GP for the same Ebolavirus species, only one *Epomophorus* bat collected in Mangina (MG) in the North-Kivu province, tested positive for Sudan virus (SUDV) using the mean + 4SD method (Figure 1, Table 3). As shown in Table 3, reactivity to glycoprotein antigens of the different Ebolavirus species was highest, ranging between 0.2% and 0.7% to 4.0% and 5.7% for Ebola (EBOV), Sudan (SUDV) or Bundibugyo (BDBV). None of the samples reacted with GP from Reston virus (RESTV). Reactivities were lower with the VP40 antigens and weakest with nucleoprotein (Table 3). In addition, simultaneous reactivity to the same antigen from different virus lineages was observed for the GPs for 0.4% to 5.2% (4–48) of samples (data not shown). Another simultaneous reactivity to at least 2 antigens from the same virus lineage was observed only for the SUDV lineage using the less stringent mean+4SD method; i.e., two *Rousettus aegyptiacus* and two *Eidolon helvum* were reactive to both GP and VP40. All insectivorous bat samples were negative using both cutoffs for all antigens.

Because the simultaneous presence of antibodies against NP and GP recombinant proteins might be too stringent as criteria, we also examined the number of samples that were reactive with at least one antigen from one of the Ebolavirus species. A total of eleven (1.4%) to 100 (12.3%) samples from frugivorous bats belonging to 4 and 6 species tested positive for a least one antigen (Table 4, Supplementary Table S3). Reactive samples were observed in the four sites studied; 2 (2%) to 8 (8.2%) in Kikwit, 1 (0.3%) to 8 (2.8%) at the Equateur Province, 3 (0.8%) to 39 (10.5%) in the Eastern region and 5 (3%) to 45 (26.8%) in Boende. The reactive samples were from fruit bat species that were previously documented to harbor Ebola antibodies, i.e., *Eidolon helvum, Epomophorus* sp., *Epomops franqueti*, *Micropteropus pusillus*, *Myonycteris torquata*, *Rousettus aegyptiacus* (Table 4). Due to the heterogeneity of species per site and differences in numbers per site, it is difficult to draw meaningful comparisons and conclusions about whether antibody levels may differ between sites and sample collection period.

## 3. Discussion

Although there is evidence for the involvement of bats in the ecology of Ebola viruses, their exact role is still not elucidated. Several studies reported presence of antibodies but only one study in Gabon identified viral RNA in addition to antibodies in a handful of animals from three species of fruit bats [13]. To clarify the meaning of Ebola virus antibodies, it is essential to document the extent to which viral RNA and shedding can be detected in species with antibodies.

In order to increase our chances to identify viral RNA in bats, we focused our efforts on studying bats as early as possible during outbreaks and tested samples from 741 bats collected during two recent EVD outbreaks and 266 from two regions that have already experienced EVD outbreaks in DRC. Overall, samples were thus collected from 1007 bats, for 903 the species identification was confirmed by sequence analyses, swabs of 676 bats were tested by RT-PCR for the presence of viral RNA, and 925 bats were tested for the presence of antibodies to Ebolavirus. We also focused on frugivorous bats because previous studies on more than 8000 bats sampled in Africa, showed higher rates of Ebolavirus antibodies in frugivorous than in insectivorous bats. Today, antibodies to Zaire Ebolavirus have been detected in eight species of frugivorous bats (*E. franqueti, H. monstrosus, M. torquata, E. helvum, E. gambianus, R. aegyptiacus, M. pusillus, L. angolensis)* and only in one genus (*Mops* sp) of insectivorous bats [13,16,37,38,39,40,50]. Unfortunately, we were unable to document the presence of viral RNA in oral and/or rectal swabs from the 604 frugivorous and 72 insectivorous bats sampled during two EVD outbreaks.

Our study includes 197 samples from the three species previously shown to harbor viral RNA, i.e.; *Epomops franqueti* (n = 160), *Myonycteris torquata* (n = 31) and *Hypsignathus monstrosus* (n = 6). Compared to the study conducted during EVD outbreaks in 2003 in Gabon and the Republic of Congo, the total number of samples that we tested here is comparable for *E. franqueti* (160 versus 117 in Gabon), but lower for *H. monstrosus* (6 versus 21) and *M. torquata* (31 versus 141) [13]. Unlike studies in Gabon, where viral RNA was detected in 4.6% (13/279) of the samples from these three species, all of our 197 samples were negative. It is important to note that oral and rectal swabs were tested from bats that were released after sampling, while in the study from Gabon, organs (i.e., liver, spleen, kidney.) of euthanized bats were tested [13]. It is most likely that viral loads are lower in swabs than in organs and influence the result of the PCR tests. So far, infectious ebolavirus has never been isolated from bats.

Ebolaviruses are most likely cleared from their hosts and can therefore only be detected for a limited period of time. Despite the fact that we collected samples during outbreaks, sampling started several weeks after the zoonotic transmission events to the index case, either directly from bats to humans or via an intermediate mammal host. It is therefore possible that the bats sampled in the study have already cleared their viruses. For example, experimental inoculation of *R. aegyptiacus* bats with Zaire Ebolavirus showed antibody development but the detection of viral RNA or shedding was infrequent [36]. In contrast, inoculation of the same species with the Marburg virus showed viremia in organs and oral and rectal shedding. The study showed also that MARV can be horizontally transmitted between bats through direct or indirect contact with infectious body fluids [35,36,51]. This also suggests that the virus may be transmitted to other animals, including humans, by the same mechanisms.

It is also important to note that in the study conducted by Leroy and colleagues in Gabon, no single bat was simultaneously positive for antibodies and RT-PCR [13]. This strengthens the case that active viral replication leading to PCR positivity is transient in infected bats and that virus is quickly cleared, with minimal, if any, overlap with seropositivity. Although, in the study of Forbes et al. on *Mops* bats in Kenya, antibodies to EBOV VP40 antigen were detected in bats that tested positive by PCR for the presence of Bombali virus [52]. Whether the rates of viral replication and infectivity differ between the different bat species in which antibodies to Ebola viruses have been identified needs to be further investigated.

The presence of Ebola antibodies confirms that bats are able to handle infection without serious signs of disease, which has already been experimentally documented, as well as for MARV [36,53]. Despite their remarkable immunity, in the case of Ebolavirus, it is still questionable whether bats are the natural host or if they are maybe an intermediate host. It would thus be worth studying the bat biology more in detail and consider for example signs of the Ebolaviruses in their food or interaction with other susceptible mammals, as putative sources of the presence of ebolavirus in bats. 

Our study focused on sampling sessions in areas during outbreaks or with previous outbreaks. We did not take into account the potential variation of virus infection and shedding over the seasons and reproduction period. For example, a study on MARV demonstrated pulses of virus circulation in juvenile Egyptian fruit bats correlating to breeding cycles. Juvenile bats are thus the predominant animals within a population that are susceptible to new infections, and significant increases in MARV infection have been demonstrated within this naïve group [19]. In contrast, the majority of bats in our survey were adults (97.4%) potentially reducing the likelihood to detect virus.

Studying antibodies provides information on a previous acute infection. Using our previously defined positivity criteria, i.e., the simultaneous presence of antibodies against the GP and NP proteins, we only observe one bat (0.2%) out of the 575 frugivorous bats tested for antibodies in outbreak areas that were positive for Sudan Ebolavirus, while the Zaire Ebolavirus was responsible for the current outbreaks. The sample was collected in the North Kivu province. In our previous studies using the same serological assay, we showed that the extent of antibodies to Ebolaviruses varied among species. The highest rates were observed in *E. helvum*, *H. monstrosus*, *M. pusillus* and *R. aegyptiacus*. Except for *M. pusillus* (n = 124), we only had few samples of these species in outbreak areas, 19 *E. helvum*, 6 *H. monstrosus*, and 5 *R. aegyptiacus*, thus reducing the likelihood to identify positive samples. Nevertheless, we identified positive antibody samples in *E. helvum* in our previous study in the western part of DRC and in other areas in Cameroon and Guinea where no EVD outbreak has been documented, illustrating that Ebolaviruses can circulate over a large geographic area across Africa between EVD outbreaks [40]. Previous studies have also shown antibodies against the Sudan virus in several species of frugivorous bats [39,40].

Although 12 of the 31 documented EVD outbreaks occurred in DRC, there are still a relatively limited number of studies reporting Ebola viruses in bats in the country. With this new study, more than 3000 samples from bats have now been studied in DRC at eight different areas with seven that are in locations with known outbreaks [40,41,42,43]. In all studies, insectivorous bats were negative for antibodies or viral RNA when tested. Unfortunately, the early studies conducted after original outbreaks around Tandala and Yambuku in 1979 and 1980 and in Kikwit in 1995, only collected a small number of frugivorous bats; 26/426 and 123/539 and all were negative for antibodies and/or virus isolation [41,42]. We report here the first study which tested, by molecular and serological assays, a large number of bats collected during ongoing EVD outbreaks in DRC. Today, only a few other studies have been conducted during outbreaks, i.e., one in Gabon and one in southeast Guinea, where the west African outbreak started [13,54].

These studies in DRC and in other areas of Africa illustrate the difficulty of documenting the role of bats as a reservoir of Ebolaviruses and their role in the ecology of these viruses. The challenges relate to the difficulties of sampling bats in their natural environment, identifying viral RNA in a bat since it is very likely that bats can clear the virus and to the different assays used for antibody testing and to the different antibody detection tests and positivity criteria used. By analogy with observations in EVD survivors, we used the simultaneous presence of antibodies against NP and GP as a criterion of positivity in order to increase specificity as described previously [40]. However, it cannot be excluded that this criterion is too strict, because the dynamics of antibodies directed against different antigens is not known in bats.

Future studies should probably focus on regular surveys of bat colonies suspected of being infected in order to increase the likelihood of detecting viral RNA. The longitudinal following may be even more relevant regarding the previously described seasonality of MARV, another filovirus [19]. Given the increasing frequency of EVD outbreaks and their potential impact, studies on the ecology and animal reservoir of Ebolaviruses are now urgently needed.

## 4. Materials and Methods

### 4.1. Collection Sites

Samples were collected in four provinces between February 2018 and October 2019 from free-ranging bats in various locations during ongoing EVD outbreaks and in areas where outbreaks have already been documented in DRC. Samples were collected as described previously [40]. Briefly, bats were captured using mist nets or harp traps in roosting and foraging sites. Bats were released immediately after sampling for ethical and conservation purposes. Dried blood spots (DBS), rectal and oral swabs were collected and preserved as described previously [40,55]. Samples were stored in the field at ambient temperature then at –20 °C in the laboratory. Data on capture sites (GPS coordinates, ecological environment), capture method, morphology (body measurements, weight, color), sex, age class (adult, juvenile) and visual identification of species were recorded for each bat sampled.

Permission to conduct research and to collect samples was obtained from the Ministry of Health and Ministry of Environment and the national ethics committee from the DRC (ESP/CE/009/2016).

### 4.2. Screening for Ebola Virus Antibodies

Dried blood spots (DBS) were tested with a Luminex-based serological assay adapted for bats as previously described, with minor modifications [40]. The assay included a total of 10 recombinant Ebola virus proteins, i.e., glycoprotein (GP), nucleoprotein (NP), or viral protein 40 (VP40) for four Ebola virus species: Zaire (EBOV), Sudan (SUDV), Bundibugyo (BDBV) and Reston (RESTV). The Taï Forest Ebola species was not included in the assay due to a lack of available recombinant protein. Briefly, whole blood from DBS was reconstituted as previously described [40,56] and 100 μL of sample adjusted at a final plasma dilution of 1:2000, taking into account the hematocrit, were incubated with 50 μL of magnetic beads coated with recombinant protein (2 μg protein/1.25 × 106 beads) in 96-well flat-bottom chimney plates (Greiner bio one, Frickenhausen, Germany) on a plate shaker at 300 rpm for 16 h at 4 °C in the dark. After washing, 0.1 μg/mL of goat anti-bat biotin-labeled IgG (Euromedex, Souffelweyersheim, France) was added to each well and incubated for 30 min at 300 rpm at room temperature. After washing, we added 50 μL of 4 μg/mL streptavidin-R-phycoerythrin (Fisher Scientific/Life Technologies, Illkirch, France) per well and incubated for 10 min at 300 rpm at room temperature. Reactions were read with BioPlex-200 (BioRad, Marnes-la-Coquette, France) or MagPix (Luminex, Austin, TX, USA). At least 100 events were read for each bead set, and results were expressed as median fluorescence intensity (MFI) per 100 beads. Samples that showed positive signals were repeated in order to validate results.

### 4.3. Cut-Off Determination

For each of the 10 antigens, we first used the general formula which used the MFI values of negative control samples as described previously, i.e., mean of 145 negative samples plus 4 × SD (standard deviation) [40]. Negative control samples were from insectivorous and frugivorous bat species born in captivity in Europe [40]. Second, we determined consensus cutoffs as the mean of three cutoffs obtained using three previously described statistical methods used in the absence of well-documented positive controls [16,40,56,57]. The cutoffs were calculated using the same methods as those described by De Nys et al. [40] and are described in details in Supplementary material 4; i.e., change point analysis method and fitted univariate binomial and exponential distributions to define the cutoff at a 0.001 risk for error. For these calculations, we used a larger panel of 8741 bats from Guinea, DRC and Cameroon, which included the samples from our previous study [40] and samples from subsequent surveys in the same countries. The large number of bat samples used for the cutoff calculation allowed the robustness of the statistical methods (Supplementary Table S2, Supplementary material 4). Analyses were performed with R version 4.0.2 software (https://www.r-project.org/, accessed on 30 March 2021). We considered a sample reactive with an antigen if the MFI values were above the cutoff value. We defined the positivity of Ebola virus antibodies as reactivity to glycoprotein (GP) and nucleoprotein (NP) of the same lineage, as in our previous studies [40,56,58].

### 4.4. Molecular Confirmation of Bat Species

The species identification recorded in the field was confirmed at the molecular level on a subset of bats using DNA extracted from DBS as described in our previous studies [40,55]. Briefly, a fragment of approximately 800 base pairs (bp) of the mitochondrial cytochrome b (cytb) was amplified using primers adapted from Irwin et al. [59]. In addition, for certain species of bats, primers targeting the 12 S region of ribosomal RNA were used [60]. PCR products were directly sequenced on an ABI 3500 sequencer (Applied Biosystems, Courtaboeuf, France). Sequences were submitted to NCBI for BLAST analysis to identify the most similar bat species. For sequences with no or low similarity (<97%) hits with species in Genbank, phylogenetic tree analysis with reference sequences was performed using maximum likelihood methods with RAxMLv8 [61] implemented in MegAlignPro version 17.2 (DNASTAR. Madison, WI, USA) in order to determine the genus. At least one sample per species and per sampling event was confirmed at the molecular level. Species identification was then extrapolated to the remaining samples by combining the molecular and morphological data records in the field. We also used morphological details on the forearm and weight measurements to distinguish *Epomophorus* sp. and *Micropteropus pusillus*, as previously documented [55,62].

### 4.5. Nucleic Acid Extraction, RT-PCR Screening and Nanopore Sequencing for Detection of Filoviruses

Total DNA and RNA were extracted from oral and rectal swabs using the NucliSens MiniMAG^®^ system (Biomerieux, Marcy-l’Etoile, France). Briefly, 250 µL of RNAlater swab sample was incubated with 1 mL of lysis buffer for 10 min and extraction was performed using manufacturer’s instructions. cDNA was first synthesized from denatured RNA using the Reverse Transcription System kit with random primers (Promega, Madison, WI, USA), following the manufacturer’s instructions. PCR screening was performed with a broadly reactive semi-nested PCR targeting a 630 bp fragment of the L gene using degenerated primers that detect *Filoviridae* family level, as previously described [15,32]. Briefly, cDNA was amplified using the GoTaq Hot Start Master Mix PCR kit (Promega, Madison, WI, USA) as follows for first and second PCR rounds: 10 cycles of 92 °C for 20 s, 50 °C for 30 s with –0.5 °C/cycle and 72 °C for 1 min, 35 cycles of 92 °C for 20 s, 50 °C for 30 s and 72 °C for 1 min. After agarose gel electrophoresis, PCR products of the expected size were sequenced by Nanopore sequencing technology. Briefly, PCR products were barcoded and pooled using the Native Barcoding Kit (Oxford Nanopore Technologies, Oxford, UK). Sequencing libraries were generated from the barcoded products using the Genomic DNA Sequencing Kit EXP-NBD104-114/SQK-LSK109 (Oxford Nanopore Technologies) and were loaded onto an R9.4 flow cell. Base-calling, adapter removal and demultiplexing were achieved using MinKnow software, and the obtained Fastq sequences were analyzed using Nanopipe pipeline (http://www.bioinformatics.uni-muenster.de, accessed on 30 March 2021), and compared to other Filovirus genus references. Additionally, samples with bands of expected size with universal primers were also tested using a previously described Ebola Zaire specific PCR assay targeting a 184 bp fragment on the VP35 gene [5].

## Figures and Tables

**Table 1:** Details and numbers of species that were collected at each site. Two letter code of sites corresponds to codes on Figure 1, -means that no samples were collected. Sites are abbreviated as follows: BK: Bikoro; IB: Iboko; IG: Ingende; BN: Beni; BT: Butembo; KM: Komanda; MG: Mangina; KK: Kikwit; BD: Boende.

				Equateur					North-Kivu/Ituri		Kikwit		Boende	Total
	**BK**	**IB**	**IG**	**subtotal**	**BN**	**BT**	**KM**	**MG**	**subtotal**	**KK**	**subtotal**	**BD**	**subtotal**	
**FRUGIVOROUS BATS**														
**Family PTEROPODIDAE**														
*Casinycteris argynnis*	1	11	-	12	-	-	-	-	-	-	-	4	4	16
*Eidolon helvum*	2	-	3	5	2	-	2	10	14	-	-	130	130	149
*Epomophorus* sp.	2	-	-	2	185	34	4	43	266	-	-	-	-	268
*Epomops franqueti*	20	57	27	104	-	-	21	35	56	2	2	17	17	179
*Hypsignathus monstrosus*	-	-	-	-	-	-	1	5	6	-	-	5	5	11
*Lissonycteris angolensis*	-	-	-	-	23	1	2	-	26	-	-	-	-	26
*Megaloglossus woermanni*	-	4	-	4	-	-	1	-	1	5	5	1	1	11
*Micropteropus pusillus*	118	-	-	118	3	-	3	-	6	33	33	3	3	160
*Myonycteris torquata*	11	16	2	29	-	-	1	1	2	30	30	5	5	66
*Rousettus aegyptiacus*	-	-	-	-	-	1	4	-	5	-	-	-	-	5
*Scotonycteris bergmansi*	-	1	-	1	-	-	-	-	-	-	-	-	-	1
**Subtotal frugivorous bats**	154	89	32	275	213	36	39	94	382	70	70	165	165	892
**INSECTIVOROUS BATS**														
**Family EMBALLONURIDAE**														
*Taphozous mauritianus*	-	-	-	-	-	-	7	-	7	-	-	-	-	7
**Family HIPPOSIDERIDAE**														
*Hipposideros caffer*	-	-	-	-	61	-	-	-	61	-	-	-	-	61
*Hipposideros* sp.	-	-	-	-	-	-	-	-	-	1	1	-	-	1
**Family MOLOSSIDAE**														
*Chaerephon* sp.	-	-	-	-	-	-	-	-	-	6	6	1	1	7
*indeterminate*	2	-	-	2	-	-	-	-	-	-	-	-	-	2
*Mops* sp.	11	-	-	11	-	-	-	-	-	-	-	-	-	11
**Family NYCTERIDAE**														
*Nycteris arge*	-	-	-	-	-	-	-	-	-	-	-	1	1	1
*Nycteris* sp.	-	-	-	-	-	-	-	-	-	-	-	1	1	1
**Family VESPERTILIONIDAE**														
*Neoromicia nanus*	-	-	-	-	-	-	-	-	-	2	2	-	-	2
*Neoromicia* sp.	-	-	-	-	-	-	-	-	-	4	4	-	-	4
*Scotophilus dinganii*	-	-	-	-	-	-	3	-	3	14	14	-	-	17
*Scotophilus nux*	-	-	-	-	-	-	-	-	-	1	1	-	-	1
**Subtotal insectivorous bats**	13	-	-	13	61	-	10	-	71	28	28	3	3	115
**TOTAL**	**167**	**89**	**32**	**288**	**274**	**36**	**49**	**94**	**453**	**98**	**98**	**168**	**168**	**1007**

**Table 2:** Numbers of samples from each species tested for presence of viral RNA by RT-PCR as described in methods, details are shown on number of samples that were tested for oral or rectal swabs only or for both. -; not tested because not available.

	N Tested	Oral Swab	Oral+Rectal Swab	Rectal Swab
**FRUGIVOROUS BATS**				
**Family *PTEROPODIDAE***				
*Casinycteris argynnis*	12	-	-	12
*Eidolon helvum*	17	-	15	2
*Epomophorus* sp.	263	-	261	2
*Epomops franqueti*	126	2	63	61
*Hypsignathus monstrosus*	5	-	5	-
*Lissonycteris angolensis*	24	-	22	2
*Megaloglossus woermanni*	4	-	-	4
*Micropteropus pusillus*	121	-	48	73
*Myonycteris torquata*	30	-	5	25
*Rousettus aegyptiacus*	1	-	1	-
*Scotonycteris bergmansi*	1	-	-	1
**Subtotal frugivorous bats**	604	2	420	182
**INSECTIVOROUS BATS**				
**Family *EMBALLONURIDAE***				
*Taphozous mauritianus*	0	-	-	-
**Family *HIPPOSIDERIDAE***				
*Hipposideros caffer*	59	56	3	-
*Hipposideros* sp.	0	-	-	
**Family *MOLOSSIDAE***				
*Chaerephon* sp.	0	-	-	-
*indeterminate*	2	-	2	-
*Mops* sp.	11	-	-	11
**Family *NYCTERIDAE***				
*Nycteris arge*	0	-	-	-
*Nycteris* sp.	0	-	-	-
**Family *VESPERTILIONIDAE***				
*Neoromicia nanus*	0	-	-	-
*Neoromicia* sp.	0	-	-	-
*Scotophilus dinganii*	0	-	-	-
*Scotophilus nux*	0	-	-	-
**Subtotal insectivorous bats**	72	56	5	11
**TOTAL**	**676**	**58**	**425**	**193**

**Table 3:** Numbers and percentages of samples with antibodies for each Ebolavirus antigen in the Luminex assay are shown at each site and in total. Numbers and percentages are expressed as a range, corresponding to values obtained by stringent or less stringent cut-off calculations as described in methods. The assay used recombinant proteins of Nucleoprotein (NP), Viral Protein-40 (VP40) or Glycoprotein (GP) for different Ebola virus lineages: Zaire (EBOV), Sudan (SUDV), Bundibugyo (BDBV) and Reston (RESTV). GP proteins from the Mayinga (GP-M) and the Kissidougou (GP-K) strain were used for EBOV.

		Kikwit	Boende	Equateur	North Kivu/Ituri	Total
		n = 98	n = 168	n = 287	n = 372	n = 925
		N (%) pos	N (%) pos	N (%) pos	N (%) pos	N (%) pos
**EBOV**	NP	0-0 (0-0)	0-0 (0-0)	1-1 (0.3-0.3)	0-0 (0-0)	1-1 (0.1-0.1)
	GP-K	1-3 (1-3)	1-21 (0.6-30.9)	0-4 (0-1.4)	3-19 (0.8-5.1)	5-47 (0.5-5.1)
	GP-M	0-4 (0-4)	1-8 (0.6-4.8)	0-2 (0.0.7)	2-7 (0.5-1.9)	3-21 (0.3-2.3)
	VP40	0-0 (0-0)	0-4 (0.0-2.4)	0-2 (0.0.7)	0-0 (0-0)	0-6 (0-0.6)
	**NP+GP**	**0-0 (0-0)**	**0-0 (0-0)**	**0-0 (0-0)**	**0-0 (0-0)**	**0-0 (0-0)**
**SUDV**	NP	0-1 (0-1)	0-0 (0-0)	0-1 (0-0.3)	1-2 (0.3-0.5)	1-4 (0.1-0.4)
	GP	0-3 (0-3)	4-42 (2.4-25)	0-3 (0-1.1)	3-5 (0.4-1.3)	7-53 (0.7-5.7)
	VP40	0-2 (0-2)	0-1 (0-0.6)	0-1 (0-0.3)	0-4 (0-1.1)	2-8 (0.2-0.8)
	**NP+GP**	**0-0 (0-0)**	**0-0 (0-0)**	**0-0 (0-0)**	**0-1 (0-0.3)**	**0-1 (0-0.1)**
**BDBV**	GP	0-2 (0-2)	1-18 (0.6-10.7)	0-3 (0-1.1)	1-14 (0.3-3.7)	2-37 (0.2-4.0)
	VP40	0-0 (0-0)	0-0 (0-0)	0-0 (0-0)	0-0 (0-0)	0-2 (0-0.2)
**RESTV**	GP	0-0 (0-0)	0-0 (0-0)	0-0 (0-0)	0-0 (0-0)	0-0 (0-0)

**Table 4:** Numbers and percentages of samples with antibodies to at least one Ebolavirus antigen in the Luminex assay are shown for each species at each site and in total. Numbers and percentages are expressed as a range, corresponding to values obtained by stringent or less stringent cut-off calculations as described in methods. na, means not applicable because no samples of the species were collected at the given site. Details on reactivity to each Ebola antigen are provided in the Supplementary Table S3.

	Equateur		North Kivu/Ituri		Kikwit		Boende		Total	
	N Tested	N (%) pos	N Tested	N (%) pos	N Tested	N (%) pos	N Tested	N (%) pos	N Tested	N (%) pos
**FRUGIVOROUS BATS**										
**Family *PTEROPODIDAE***										
*Casinycteris argynnis*	11	0 (0)	na		na		4	0 (0)	15	0 (0)
*Eidolon helvum*	5	0 (0)	14	2–6 (14.3–42.9)	na		130	5–44 (3.8–33.8)	149	7–50 (4.7–33.6)
*Epomophorus* sp.	2	0 (0)	187	0–22 (0–11.8)	na		na		189	0–22 (0–11.6)
*Epomops franqueti*	104	0–3 (0–2.9)	56	0–5 (0–8.9)	2	0 (0)	17	0 (0)	179	0–8 (0–4.5)
*Hypsignathus monstrosus*	na		6	0 (0)	na		5	0 (0)	11	0 (0)
*Lissonycteris angolensis*	na		26	0 (0)	na		na		26	0 (0)
*Megaloglossus woermanni*	4	0 (0)	1	0 (0)	5	0 (0)	1	0 (0)	11	0 (0)
*Micropteropus pusillus*	118	1–4 (0.8–3.4)	4	0–2 (0–50)	33	1–4 (3–12.1)	3	0 (0)	158	2–10 (1.2–6.3)
*Myonycteris torquata*	29	0–1 (0–3.4)	2	0 (0)	30	1–4 (3.3–13.3)	5	0–1 (0–20)	66	1–6 (1.5–9.1)
*Rousettus aegyptiacus*	na		5	1–4 (20–80)	na		na		5	1–4 (20–80)
*Scotonycteris bergmansi*	1	0 (0)	na		na		na		1	0 (0)
**Subtotal frugivorous bats**	274	1–8 (0.4–2.9)	301	3–39 (1–13)	70	2–8 (2.9–11.4)	165	5–45 (3–27.3)	810	11–100 (1.4–12.3)
**INSECTIVOROUS BATS**										
**Family *EMBALLONURIDAE***										
*Taphozous mauritianus*	na		7	0 (0)	na		na		7	0 (0)
**Family *HIPPOSIDERIDAE***										
*Hipposideros caffer*	na		61	0 (0)	na		na		61	0 (0)
*Hipposideros* sp.	0		na		1	0 (0)	na		1	0 (0)
**Family *MOLOSSIDAE***										
*Chaerephon* sp.	na		na		6	0 (0)	1	0 (0)	7	0 (0)
Indeterminate	2	0 (0)	na		na		na		2	0 (0)
*Mops* sp.	11	0 (0)	na		na		na			0 (0)
**Family NYCTERIDAE**										
*Nycteris arge*	na		na		na		1	0 (0)	1	0 (0)
*Nycteris* sp.	na		na		na		1	0 (0)	1	0 (0)
**Family *VESPERTILIONIDAE***										
*Neoromicia nanus*	na		na		2	0 (0)	na		2	0 (0)
*Neoromicia* sp.	na		na		4	0 (0)	na		4	0 (0)
*Scotophilus dinganii*	na		3	0 (0)	14	0 (0)	na		17	0 (0)
*Scotophilus nux*	na		na		1	0 (0)	na		1	0 (0)
**Subtotal insectivorous bats**	13	0 (0)	71	0	28	0 (0)	3	0 (0)	115	0 (0)
**Total**	**287**	**1–8 (0.3–2.8)**	**372**	**3–39 (0.8–10.5)**	**98**	**2–8 (2–8.2)**	**168**	**5–45 (3–26.8)**	**925**	**11–100 (1.2–10.8)**

## Data Availability

Data presented in this study are available upon request, and data on bat sampling are available on the EBO-SURSY website (https://rr-africa.oie.int/fr/projets/ebo-sursy-fr/, accessed on 30 March 2021).

## Supplementary Materials

The following are available online at https://www.mdpi.com/article/10.3390/pathogens10050557/s1, Table S1: Collection sites, date of collection, and number of bats sampled in the Democratic Republic of the Congo, and number of serological and molecular tests performed; Table S2: MFI cut-off values calculated with different methods as described in Methods section for the different Ebolavirus strains [Zaire (EBOV), Sudan (SUDV), Bundibugyo (BDBV) and Reston (RESTV)] and the different antigens used per virus. Table S3: Numbers of samples with antibodies to each Ebolavirus antigen, to each Ebolavirus species and simultaneous NP+GP EBOV and SUDV antigens in the Luminex assay for each tested bat species. Numbers are obtained by stringent (Mean of the cutoffs; CO 1) or less stringent cutoff (Mean + 4SD; CO 2) calculations as described in methods. Supplementary material 4: Determination of Cutoffs.
