# Supplementary material for: Investigating the Circulation of Ebola Viruses in Bats during the Ebola Virus Disease Outbreaks in the Equateur and North Kivu Provinces of the Democratic Republic of Congo from 2018
Source: Pathogens. 2021 May 4;10(5):557. doi: 10.3390/pathogens10050557 (PMC8147758; doi:10.3390/pathogens10050557)
Supplement: Supplementary file 1 [file pathogens-10-00557-s001.zip › pathogens-1186964-supplementary.pdf]

Supplementary Table S1: Collection sites, date of collection, and number of bats sampled in the Democratic Republic of the Congo, and number of serological and molecular tests performed.

Province	Site	Site name	Nb of collected bats	Date of collection	Date of outbreak	Nb of bats tested by PCR	Nb of bats tested by serology
Ongoing outbreaks							
**Equateur**			**288**			288 (100%)	287 (99.6%)
	Bikoro	BK	167	May-June 2018	outbreak May-July 2018	167	167
	Iboko	IB	89	June 2018		89	88
	Ingende	IG	32	June 2018		32	32
**East**			**453**			388 (85.6%)	372 (82.1%)
	Beni	BN	274	Dec 2018-May 2019	outbreak July 2018-june 2020	271	194
	Butembo	BT	36	March 2019		36	36
	Komanda	KM	49	Feb 2019		0*	49
	Mangina	MG	94	March 219		81	93
Past outbreaks							
**Kwilu**	Kikwit	KK	**98**	Feb 2018	past outbreak 1995	0	98 (100%)
**Tshuapa**	Boende	BD	**168**	Sept-Oct 2019	past outbreak 2014	0	168 (100%)
**Total**			**1007**			**676**	**925**

*Swab samples were not available for samples collected at this site.

Supplementary Table S2 : MFI cut-off values calculated with different methods as described in Methods section for the different Ebolavirus strains [Zaire (EBOV), Sudan (SUDV), Bundibugyo (BDBV) and Reston (RESTV)] and the different antigens used per virus.

GP-EBOV-K, GP recombinant protein derived from the EBOV Kissidougou strain from West Africa in 2014.
GP-EBOV-M, GP recombinant protein derived from the EBOV Mayinga strain from DRC in 1976.
Mean of negatives was calculated on negative control samples (n=145) that were collected from captive-born insectivorous bats (*Carollia perspicillata*, n=103) hosted at the Zoo of Montpellier, France, and from two frugivorous species (*Pteropus giganteus*, n=19; *Rousettus aegyptiacus*, n=23) from the Zoo of Stuttgart, Germany, as described in De Nys et al., 2018. The other methods were calculated on a sample set of wild captured bats from Guinee, Cameroon and DRC consisting of DBS samples from 8741 bats. Details on the statistical methods are provided in the supplementary material 4.

Antigen	Mean+4SD*	Mean of the cutoffs
NP_EBOV	**71**	**196**
GP_EBOV-K	**128**	**609**
GP_ EBOV-M	**307**	**707**
VP40_ EBOV	**75**	**177**
NP_SUDV	**131**	**258**
GP_SUDV	**251**	**1896**
VP40_SUDV	**88**	**182**
GP_BDBV	**99**	**432**
VP40_BDBV	**363**	**664**
GP_RESTV	**249**	**140**

Supplementary Table 3. Numbers of samples with antibodies to each Ebolavirus antigen, to each Ebolavirus species and simultaneous NP+GP EBOV and SUDV antigens in the Luminex assay for each tested bat species. Numbers are obtained by stringent (Mean of the cutoffs; CO 1) or less stringent cutoff (Mean + 4SD, CO 2) calculations as described in methods. - means seronegative sample. GP-EBOV-K, GP recombinant protein derived from the EBOV Kissidougou strain, GP-EBOV-M, GP recombinant protein derived from the EBOV Mayinga strain.

		EBOV								SUDV						BDBV				REBV		NP+GP reactivity			
		NP EBOV		GP-EBOV-K		GP-EBOV-M		VP40-EBOV		NP-SUDV		GP-SUDV		VP40-SUDV		GP-BDBV		VP40-BDBV		GP-REBV		NP+GP EBOV		NP+GP SUDV	
	Nb of tested bats	CO 1	CO 2	CO 1	CO 2	CO 1	CO 2	CO 1	CO 2	CO 1	CO 2	CO 1	CO 2	CO 1	CO 2	CO 1	CO 2	CO 1	CO 2	CO 1	CO 2	CO 1	CO 2	CO 1	CO 2
**Frugivorous bats**	**810**	**1**	**1**	**4**	**47**	**3**	**21**	**0**	**6**	**1**	**3**	**7**	**82**	**2**	**8**	**2**	**37**	**0**	**0**	**0**	**0**	**0**	**0**	**0**	**1**
Casinycteris argynnis	15	-	-	-	-	-	-	-	-	-	-	-	-	-	-	-	-	-	-	-	-	-	-	-	-
Eidolon helvum	149	-	-	3	27	2	12	-	3	1	1	6	47	-	2	2	23	-	-	-	-	-	-	-	-
Epomophorus sp.	189	-	-	-	8	-	2	-	-	-	1	-	19	-	1	-	6	-	-	-	-	-	-	-	1
Epomops franqueti	179	-	-	-	2	-	1	-	2	-	-	-	5	-	-	-	1	-	-	-	-	-	-	-	-
Hypsignathus monstrosus	11	-	-	-	-	-	-	-	-	-	-	-	-	-	-	-	-	-	-	-	-	-	-	-	-
Lissonycteris angolensis	26	-	-	-	-	-	-	-	-	-	-	-	-	-	-	-	-	-	-	-	-	-	-	-	-
Megaloglossus woermanni	11	-	-	-	-	-	-	-	-	-	-	-	-	-	-	-	-	-	-	-	-	-	-	-	-
Micropteropus pusillus	158	1	1	-	4	-	2	-	-	-	-	-	6	1	2	-	2	-	-	-	-	-	-	-	-
Myonycteris torquata	66	-	-	-	3	-	2	-	1	-	1	-	1	1	1	-	2	-	-	-	-	-	-	-	-
Rousettus aegyptiacus	5	-	-	1	3	1	2	-	-	-	-	1	4	-	2	-	3	-	-	-	-	-	-	-	-
Scotonycteris bergmansi	1	-	-	-	-	-	-	-	-	-	-	-	-	-	-	-	-	-	-	-	-	-	-	-	-
**Insectivorous bats**	**115**	**0**	**0**	**0**	**0**	**0**	**0**	**0**	**0**	**0**	**0**	**0**	**0**	**0**	**0**	**0**	**0**	**0**	**0**	**0**	**0**	**0**	**0**	**0**	**0**
EMBALLONURIDAE	**7**	**-**	**-**	**-**	**-**	**-**	**-**	**-**	**-**	**-**	**-**	**-**	**-**	**-**	**-**	**-**	**-**	**-**	**-**	**-**	**-**	**-**	**-**	**-**	**-**
Taphozous mauritianus	7	-	-	-	-	-	-	-	-	-	-	-	-	-	-	-	-	-	-	-	-	-	-	-	-
HIPPOSIDERIDAE	**62**	**-**	**-**	**-**	**-**	**-**	**-**	**-**	**-**	**-**	**-**	**-**	**-**	**-**	**-**	**-**	**-**	**-**	**-**	**-**	**-**	**-**	**-**	**-**	**-**
Hipposideros caffer	61	-	-	-	-	-	-	-	-	-	-	-	-	-	-	-	-	-	-	-	-	-	-	-	-
Hipposideros sp.	1	-	-	-	-	-	-	-	-	-	-	-	-	-	-	-	-	-	-	-	-	-	-	-	-
MOLOSSIDAE	**20**	**-**	**-**	**-**	**-**	**-**	**-**	**-**	**-**	**-**	**-**	**-**	**-**	**-**	**-**	**-**	**-**	**-**	**-**	**-**	**-**	**-**	**-**	**-**	**-**
Chaerephon sp.	7	-	-	-	-	-	-	-	-	-	-	-	-	-	-	-	-	-	-	-	-	-	-	-	-
INDETERMINE	2	-	-	-	-	-	-	-	-	-	-	-	-	-	-	-	-	-	-	-	-	-	-	-	-
Mops sp.	11	-	-	-	-	-	-	-	-	-	-	-	-	-	-	-	-	-	-	-	-	-	-	-	-
NYCTERIDAE	**2**	**-**	**-**	**-**	**-**	**-**	**-**	**-**	**-**	**-**	**-**	**-**	**-**	**-**	**-**	**-**	**-**	**-**	**-**	**-**	**-**	**-**	**-**	**-**	**-**
Nycteris arge	1	-	-	-	-	-	-	-	-	-	-	-	-	-	-	-	-	-	-	-	-	-	-	-	-
Nycteris sp.	1	-	-	-	-	-	-	-	-	-	-	-	-	-	-	-	-	-	-	-	-	-	-	-	-
VESPERTILIONIDAE	**24**	**-**	**-**	**-**	**-**	**-**	**-**	**-**	**-**	**-**	**-**	**-**	**-**	**-**	**-**	**-**	**-**	**-**	**-**	**-**	**-**	**-**	**-**	**-**	**-**
Neoromicia nanus	2	-	-	-	-	-	-	-	-	-	-	-	-	-	-	-	-	-	-	-	-	-	-	-	-
Neoromicia sp.	4	-	-	-	-	-	-	-	-	-	-	-	-	-	-	-	-	-	-	-	-	-	-	-	-
Scotophilus dinganii	17	-	-	-	-	-	-	-	-	-	-	-	-	-	-	-	-	-	-	-	-	-	-	-	-
Scotophilus nux	1	-	-	-	-	-	-	-	-	-	-	-	-	-	-	-	-	-	-	-	-	-	-	-	-
**TOTAL**	**925**	**1**	**1**	**4**	**47**	**3**	**21**	**0**	**6**	**1**	**3**	**7**	**82**	**2**	**8**	**2**	**37**	**0**	**0**	**0**	**0**	**0**	**0**	**0**	**1**

Supplementary material 4 : Determination of Cutoffs

In the absence of positive control samples, we used 4 different statistical methods to determine the MFI cutoff value for each antigen [1,2]. First, we used a general formula that involved the MFI of the 145 negative control samples, and we assigned the cutoff as mean plus 4 times the SD (mean + 4×SD). We collected negative control samples (n = 145) from a captive-born bats [3].

Second, we determined consensus cutoffs as the mean of three cutoffs obtained using three previously described statistical methods [3]. For these calculations, we used a larger panel of 8741 bats from Guinea, DRC and Cameroon, that included the samples from our previous study [3] and samples from subsequent surveys in the same countries. The large number of bat samples used for the cutoff calculation allowed the robustness of the statistical methods. Thus we decided to take the mean of the cut-off using the following 3 calculated cut-offs. Briefly, for the first method, we used a change point analysis [4] to identify the value at which statistical properties of the underlying probability distribution changed. This value was used to identify outliers and classify them as reactive. We used the R package changepoint [5] to calculate a single shift in the arithmetic mean with the at-most-1-change method [6]. Secondly, we fitted univariate distributions to our data and defined the cutoff as a 0.001 risk for error, as was used in other virus serology studies [7,8]. We reduced the set of candidate distributions following a bootstrapped skewness-kurtosis analysis [9]. We performed fitting by maximum-likelihood estimation and selected the best-fit distribution on the basis of the Akaike information criteria with the R library fitdistrplus [10]. A negative binomial distribution best-fit the data; however, we also used as a third method, the negative exponential distribution as in Pourrut et al. and Laing et al. [7,8]. For every antigen, we computed bootstrap values using 10,000 replicates and averaged.

We performed analyses with R version 4.0.2. software (https://www.r-project.org/). We considered a blood sample reactive if the MFI of the reaction was above the cutoff. We defined Ebola virus antibody positivity as reactivity to glycoprotein and nucleoprotein of the same lineage, as was done in our previous study [11].

MFI cut-off values calculated with different methods as described in Methods section and supplementary table S2, for the different Ebolavirus strains [Zaire (ZEBOV), Sudan (SUDV), Bundibugyo (BDBV) and Reston (RESTV)] and the different antigens used per virus.

Antigen	Mean+4SD *[1]	mean of the cutoffs*[2]	Changepoint method*[2]	Exponential method (0.01) *[2]	Binomial method (0.01) *[2]
NP_ZEBOV	**71**	**196**	396	129	62
GP_ZEBOV-K	**128**	**609**	386	1052	389
GP_ ZEBOV-M	**307**	**707**	427	1250	444
VP40_ ZEBOV	**75**	**177**	361	109	61
NP_SUDV	**131**	**258**	417	253	103
GP_SUDV	**251**	**1896**	1046	3458	1185
VP40_SUDV	**88**	**182**	241	196	110
GP_BDBV	**99**	**432**	367	666	262
VP40_BDBV	**363**	**664**	1749	166	77
GP_RESTV	**249**	**140**	233	132	55

*[1] calculated on a panel of 145 samples
*[2] calculated on a panel of 8741 samples

References

1. Gilbert, A.T.; Fooks, A.R.; Hayman, D.T.S.; Horton, D.L.; Müller, T.; Plowright, R.; Peel, A.J.; Bowen, R.; Wood, J.L.N.; Mills, J.; et al. Deciphering Serology to Understand the Ecology of Infectious Diseases in Wildlife. *Ecohealth* **2013**, *10*, 298–313, doi:10.1007/s10393-013-0856-0.
2. Peel, A.J.; McKinley, T.J.; Baker, K.S.; Barr, J.A.; Crameri, G.; Hayman, D.T.S.; Feng, Y.-R.; Broder, C.C.; Wang, L.-F.; Cunningham, A.A.; et al. Use of Cross-Reactive Serological Assays for Detecting Novel Pathogens in Wildlife: Assessing an Appropriate Cutoff for Henipavirus Assays in African Bats. *J. Virol. Methods* **2013**, *193*, 295–303, doi:10.1016/j.jviromet.2013.06.030.
3. De Nys, H.M.; Kingebeni, P.M.; Keita, A.K.; Butel, C.; Thaurignac, G.; Villabona-Arenas, C.-J.; Lemarcis, T.; Geraerts, M.; Vidal, N.; Esteban, A.; et al. Survey of Ebola Viruses in Frugivorous and Insectivorous Bats in Guinea, Cameroon, and the Democratic Republic of the Congo, 2015-2017. *Emerging Infect. Dis.* **2018**, *24*, doi:10.3201/eid2412.180740.
4. Lardeux, F.; Torrico, G.; Aliaga, C. Calculation of the ELISA's Cut-off Based on the Change-Point Analysis Method for Detection of Trypanosoma Cruzi Infection in Bolivian Dogs in the Absence of Controls. *Mem. Inst. Oswaldo Cruz* **2016**, *111*, 501–504, doi:10.1590/0074-02760160119.
5. Killick, R.; Eckley, I. Changepoint:An R Package for Changepoint Analysis. *Journal of Statistical Software* **2014**, *58*, 1–19.
6. Hinkley, D.V. Inference about the Change-Point in a Sequence of Random Variables. *Biometrika* **1970**, *57*, 1–17, doi:10.1093/biomet/57.1.1.
7. Pourrut, X.; Souris, M.; Towner, J.S.; Rollin, P.E.; Nichol, S.T.; Gonzalez, J.-P.; Leroy, E. Large Serological Survey Showing Cocirculation of Ebola and Marburg Viruses in Gabonese Bat Populations, and a High Seroprevalence of Both Viruses in Rousettus Aegyptiacus. *BMC Infectious Diseases* **2009**, *9*, 159, doi:10.1186/1471-2334-9-159.
8. Laing, E.D.; Mendenhall, I.H.; Linster, M.; Low, D.H.W.; Chen, Y.; Yan, L.; Sterling, S.L.; Borthwick, S.; Neves, E.S.; Lim, J.S.L.; et al. Serologic Evidence of Fruit Bat Exposure to Filoviruses, Singapore, 2011-2016. *Emerging Infect. Dis.* **2018**, *24*, 114–117, doi:10.3201/eid2401.170401.
9. Cullen, A.C.; Frey, H.C.; Frey, C.H. *Probabilistic Techniques in Exposure Assessment: A Handbook for Dealing with Variability and Uncertainty in Models and Inputs*; Springer Science & Business Media, 1999; ISBN 978-0-306-45957-3.
10. Delignette-Muller, M.L.; Dutang, C. Fitdistrplus: An R Package for Fitting Distributions. *Journal of Statistical Software* **2015**, *64*, 1–34, doi:10.18637/jss.v064.i04.
11. Ayouba, A.; Touré, A.; Butel, C.; Keita, A.K.; Binetruy, F.; Sow, M.S.; Foulongne, V.; Delaporte, E.; Peeters, M. Development of a Sensitive and Specific Serological Assay Based on Luminex Technology for Detection of Antibodies to Zaire Ebola Virus. *Journal of Clinical Microbiology* **2017**, *55*, 165–176, doi:10.1128/JCM.01979-16.
